# Novel Television-Based Cognitive Training Improves Working Memory and Executive Function

**DOI:** 10.1371/journal.pone.0101472

**Published:** 2014-07-03

**Authors:** Evelyn Shatil, Jaroslava Mikulecká, Francesco Bellotti, Vladimír Bureš

**Affiliations:** 1 CogniFit Inc., New York, New York, United States of America; 2 Department of Psychology and the Center for Psychobiological Research, Max Stern Academic College of Emek Yezreel, Yezreel Valley, Israel; 3 Faculty of Informatics and Management, University of Hradec Králové, Hradec Králové, Czech Republic; 4 Department of Electrical, Electronic, Telecommunications Engineering and Naval Architecture, University of Genoa, Genoa, Italy; University of Leicester, United Kingdom

## Abstract

The main study objective was to investigate the effect of interactive television-based cognitive training on cognitive performance of 119 healthy older adults, aged 60–87 years. Participants were randomly allocated to a cognitive training group or to an active control group in a single-blind controlled two-group design. Before and after training interactive television cognitive performance was assessed on well validated tests of fluid, higher-order ability, and system usability was evaluated. The participants in the cognitive training group completed a television-based cognitive training programme, while the participants in the active control group completed a TV-based programme of personally benefiting activities. Significant improvements were observed in well validated working memory and executive function tasks in the cognitive training but not in the control group. None of the groups showed statistically significant improvement in life satisfaction score. Participants' reports of “adequate” to “high” system usability testify to the successful development and implementation of the interactive television-based system and compliant cognitive training contents. The study demonstrates that cognitive training delivered by means of an interactive television system can generate genuine cognitive benefits in users and these are measurable using well-validated cognitive tests. Thus, older adults who cannot use or afford a computer can easily use digital interactive television to benefit from advanced software applications designed to train cognition.

## Introduction

With increasing age crystallised intelligence (the ability to acquire and store general and cultural knowledge) is believed to rise while fluid intelligence (the ability to observe complex relationships and to infer or predict subsequent relationships or actions on the basis of those observations) is known to decline, starting already in the middle of the fourth decade of life [1], [2]. Computerised cognitive training is widely available for the preservation of cognitive function in older adults [3], [4], [5] but computer and internet use decrease sharply with age, even in affluent countries [6], [7], [8]. Social, financial, cognitive, or psychological barriers [6], [9] impede seniors' use of computer-based information technology, and increased stress and decreased self-efficacy loom large in their interactions with computers [10]. Television (TV) via a TV set, however, remains by far the most popular medium viewed by Europeans [11] and the elderly report being most comfortable with it [12]. Interactive TV (iTV) provides users with new TV viewing experiences. Interactivity in iTV can be defined as simply being anything that takes the user beyond passive watching and lets the user make choices and take action [13]. Thus iTV can be considered as an alternative to computer for cognitive training delivery representing cheap alternative based on technology already available in households and most elderly citizens are familiar with it. Moreover, it does not usually engender fear and awe as computers might [14].

Therefore, using the iTV cognitive training, we attempted to enhance fluid cognitive abilities such as working memory (the ability to hold and manipulate novel, transient information), executive control (the ability to regulate and monitor thought and action) and analogical reasoning (the ability to identify analogical relationships and to draw inferences on the basis of that comprehension). In the two years that preceded the study reported here an iTV system, equipped with TV-compatible cognitive training content was designed, tested and operated that enabled older adults, who cannot use or afford a computer, to engage in cognitive training activities. To design the iTV system and compliant applications and to run the experiment reported here, eight academic and commercial institutions collaborated within the FP7 framework of the European Union (see Table 1). The iTV system design fulfilled requirements of open development supporting testing in lab and home settings; prompt system-effectiveness feedback; application use independently of underlying TV stream and seamless transportation of users' data on different TV sets. The system consisted of an iTV Philips simulator, a flash disk containing applications and data, a digital TV set, and two cursor-moving devices: a novel pointer and a remote control. The simulator replicated a TV set-top box (STB) in terms of memory, computation power and graphics and enabled efficient designing, running and verifying the iTV applications in a single platform. The cognitive training programme was developed by CogniFit, Israel, and included twenty one cognitive tasks, including divided attention, focus, planning, updating, monitoring, inhibition, memory, perception, and naming. The TV compatible software for this programme was developed by the University of Genoa, Italy. This software managed, stored, and visually displayed user interaction data in real time, the Individualised Training System, was responsible for scheduling the tasks according to the actual user profile and allowed the user to interact with the tasks.

**Table 1:** Members of the Vital Mind consortium.

Name of Institution or Business	Responsibility
Cognifit Ltd., Yokneam Illit, Israel	Coordination of the project, design and testing of cognitive content.
Goldsmiths' College, London, United Kingdom	Dissemination, exploitation, standards and ethics.
Philips Consumer Lifestyle B.V. Eindhoven, Netherlands	Design, development and testing of user interface.
Czech Technical University in Prague, Prague, Czech Republic	Design, implementation and testing of content (applications Family Tree, Physical Exercises and Training and User Error Rate Evaluation).
Universita degli Studi di Genova, Genoa, Italy	VM technology design and cognitive content implementation. Design and implementation of the Family Screen Composer application.
The University of Dundee, Dundee, United Kingdom	Experimental evaluation of user interface technology, design of cognitive content verification.
University of Hradec Kralove, Hradec Kralove, Czech Republic	Cognitive content and technology verification.
Czech Television, Prague, Czech Republic	iTV system verification.

## Research Objective

Unlike computerised cognitive training, which is widely used and researched, iTV cognitive training is a brand new concept. To our knowledge, the only such existing system in the world is the system we developed in this project and used for this study. The goal of this study was, therefore to verify whether iTV-delivered cognitive training would engender genuine and measurable benefits in cognitive function, specifically in fluid cognition. To answer this question we trained and subsequently measured higher-order processing general abilities such as working memory, executive control and analogical reasoning.

## Methods

The study was undertaken with the understanding and written informed consent of each participant and according to the World Medical Association Declaration of Helsinki principles. Since the research was conducted in the FP7 Framework, the Ethical Roadmap Report was created and followed.

### Participants and design

The efficacy of iTV-delivered cognitive training was tested in a controlled experiment with 140 participants randomly assigned to an equal sized cognitive training or a control group. The study participants were recruited through advertising and by invitation to presentations. All were told that they would engage in cognitive enhancement activities and provided informed consent. Individuals who scored <27 on MMSE or had corrected vision below 20/40 were excluded. Researchers were aware of group assignment. Except for 5 couples in the cognitive training group who trained at home, all study participants trained at the university laboratories. Cognitive group participants who trained at home were similar to those who trained in the lab on age, education and sex distribution (t = −1.23, p = .22, t = −.036, p = .97 and Chi-Square = 2.83, p = .09, respectively). These subjects were included in the main analysis, but their cognitive outcome data was also used to verify the feasibility of home-based TV-delivered cognitive training. Both interventions were designed to be iTV compliant, lasted eight weeks and were delivered weekly in three 20 minute sessions, each containing 3 different activities. Demographic, cognitive and life satisfaction information was collected at the onset of the study for screening and group-equating purposes, and paper and pencil standardised cognitive tests were administered at the university laboratories before and after the intervention. Baseline and post-training scores were compared between and within the groups. System usability was evaluated at the end of the study using a scaled judgement statement.

### Materials

#### i. The interventions

For cognitive training, CogniFit's tests, validated with healthy seniors [15], [16] [17] and with individuals with diagnosed neurological conditions [18], [19], [20], [21], [22], was used. For each trainee and for each session, three of 21 training exercises were selected and assigned, based on the results of an initial neurocognitive evaluation, the CogniFit Neuropsychological Evaluation [23], [24]. As training proceeded, a performance analysis mechanism continually adapted the exercises difficulty levels. See Cognitive Training Programme S1 for a description of the cognitive training exercises. Control group participants engaged in three non-cognitive applications: they composed family stories using memories of life milestones such as weddings, births, travels; digitalised personal photographs to build family trees and performed physical exercises based on Mind Jogging [25].

#### ii. Personal and demographic attributes at baseline

*Quality of Life (SEIQoL)* is a questionnaire that allows people to designate areas of life which are most important to them, rate their level of satisfaction with each, and indicate the relative importance of each to their overall quality of life [26], [27]. The SEIQOL index is then computed as a weighted total. A score of 100 indicates the best quality of life.

*Mini-Mental Status Examination* (*MMSE*) was used to screen for diagnosed neurological conditions [28]. A higher score denotes a better performance.

To represent *cognitive reserve*, we used a measure of the combined (additive) years of formal and informal education.

All participants held a structured diary which allowed them to enter frequency and duration of *challenging mental and physical activities during the interventions* with possible cognitive enhancement implications and not controlled by the experiment. At the end of the interventions we analysed the diaries and determined how many hours the participants had spent (during the intervention period) doing other challenging mental activities such as working with computers, learning foreign languages, attending special study programmes for seniors, reading books, being employed and doing physical activities such as walking, cycling, housekeeping, swimming, gardening.

#### iii. The outcomes

*The TONI-3* (*Test of non-verbal intelligence, 3rd Ed.*) is a major revision of the statistically reliable and validated Test of Nonverbal Intelligence [29]. TONI-3 also has the further benefit of being available in parallel forms, which means there are two versions of the test which are directly comparable with each other but which are distinct enough in their content to remove the possibility of learning or interference effects between baseline and post-training administrations.

*Trail Making Test* (TMT), parts A (TMT-A) and B (TMT-B) [30], measures executive functions, such as complex visual-motor conceptual screening, planning, organisation, abstract thinking and response inhibition [31]. Both the TMT-A and TMT-B versions, were administered.

*Digit span (DS)* is a test of working memory [32]. The forward version (DSF) of this test is based on the presentation of a sequence of individual digits starting with 2 digits. On completion of presentation, participants repeat the digit sequence. The sequence length then increases following a correct response. The test continues until the longest sequence is failed twice. The reverse version (DSR) of the test has the same procedure as the forward one, except that participants respond with the sequence of digits in reverse order. Both the forward and backward versions were administered.

*The well-being index (WHO-5)* is a short screening instrument, compiled by the World Health Organisation, for the detection of depression in the general population and provides subjective ratings of current mental & physical wellbeing [33]. It was used to measure possible indirect effects of training. Each of the five items is rated on a 6-point Likert scale from 0 ( =  not present) to 5 ( =  constantly present). The theoretical raw score ranges from 0 to 25, higher scores mean better wellbeing.

iTV *system usability* was examined using the statement “Overall, I would rate the user-friendliness of this product as: Worst Imaginable/Awful/Poor/OK/Good/Excellent/Best Imaginable”.

### Data processing

#### i. Statistical analyses

SPSS 17 [34] software package was used for statistical analyses. T-tests for independent samples and Chi-Square tests were used to determine differences in personal and demographic attributes between the two groups at baseline (Tables 2 and 3). Mixed effects models for repeated measures were used to evaluate differences within and between groups in the cognitive outcomes (DSF, DSR, Digit Span Total - DST, TMT-A, TMT-B, TMT Total – TMT-T, TONI-Test of non-verbal intelligence, 3rd ed., and WHO-5 Well-Being Index). A separate model was established for each variable. The models allowed us to assess differences in baseline scores between the two groups (Table 4), differences between baseline and post-training scores within each group (Tables 5 and 6), and whether any of the differences varied between the groups (Table 7). The independent variables included group (cognitive training or active control), time (baseline or post-training), and group by time interaction; the dependent variable was the cognitive outcome variable. Group and time were categorical fixed factors, with the participant being the random factor.

**Table 2:** Cognitive training and control groups' attributes at the onset of the study.

Attributes	Cognitive Training Group		Control Group		
	(N = 60)		(N = 59)		
	Mean [Range]	SD	Mean [Range]	SD	*t* statistics**
Age (in years)	67.7 [60–87]	5.8	68.3 [61–85]	5.8	0.55
Cognitive Reserve (Formal and informal education in years)	19.6 [11–43]	7.1	17.9 [11–46]	7.7	−1.25
MMSE*	28.4 [17]–[30]	2.1	28.9 [23]–[30]	1.4	1.24
SEIQoL*	73.2 [21 – 100]	17.1	77.2 [47– 100]	11.4	−1.47
Gender (N female;% female)	38; 63.3%		37; 62.7%	Chi-Square 0.55	

*MMSE = Mini-Mental State Examination, SEIQOL = Schedule for the Evaluation of Individual Quality of Life.

** None of the *t* or Chi-Square values reached statistical significance.

**Table 3:** Challenging mental and physical activities.

Cognitive diaries held by participants during the intervention period	Cognitive Training Group		Control Group		
	(N = 60)		(N = 59)		
	Mean [Range]	SD	Mean [Range]	SD	*t* statistics*
Total challenging mental activities in hours	42.1 [ 0–263]	49.9	47.2 [0 – 240]	59.9	0.47
Total physical activities in hours	165.9 [14 – 486]	109.9	150.5 [13 – 388]	80.4	−0.82

* None of the *t v*alues reached statistical significance.

**Table 4:** Means, SDs, mean differences and Fs for the two groups at baseline.

	Cognitive Training Group N = 60		Active Control Group N = 59			
	Mean	SD	Mean	SD	Mean Difference	F (df = 1.119)
DSF	5.69	1.35	5.92	1.21	−0.23	0.85
DSR	4.90	1.18	4.93	1.34	−0.03	0.99
DST	10.62	2.18	10.85	2.16	−0.23	0.28
TMT-A	48.42	19.37	46.35	13.40	2.07	0.51
TMT-B	100.23	54.78	92.76	31.21	7.47	1.03
TMT-T	148.65	69.15	139.10	41.14	9.55	0.99
TONI-3	107.05	14.75	107.80	16.83	−0.75	0.08
WHO-5	64.53	20.27	67.36	16.56	−2.83	0.71

**Table 5:** Baseline and post-training Means and SDs; mean differences and Fs for the cognitive training group.

	Cognitive Training Group					
	Baseline Mean N = 60	SD	Post-training Mean N = 60	SD	Mean Difference	F (df = 1.119)
DSF	5.69	1.35	6.87	1.48	1.18	34.07***
DSR	4.90	1.18	6.02	1.33	1.12	35.11***
DST	10.62	2.18	12.89	2.57	2.27	50.40***
TMT-A	48.42	19.37	39.85	16.35	−8.57	20.23***
TMT-B	100.23	54.78	81.90	41.63	−18.33	26.15***
TMT-T	148.65	69.15	121.75	55.86	−26.90	34.19***
TONI-3	107.05	14.75	111.77	14.16	4.72	6.58*
WHO-5	64.53	20.27	65.80	20.43	1.27	0.26

**Table 6:** Baseline and post-training Means and SDs; mean differences and Fs for the active control group.

	Active Control Group					
	Baseline Mean N = 59	SD	Post-training Mean N = 59	SD	Mean Difference	F (df = 1.119)
DSF	5.92	1.21	6.12	1.35	0.27	1.73
DSR	4.93	1.34	5.19	1.24	0.27	1.86
DST	10.85	2.16	11.36	2.35	0.52	0.10
TMT-A	46.35	13.40	41.62	14.07	−4.73	6.05*
TMT-B	92.76	31.21	85.26	29.23	−7.50	4.30*
TMT-T	139.10	41.14	126.87	39.14	−12.23	6.94*
TONI-3	107.80	16.83	108.88	12.60	1.08	0.34
WHO-5	67.36	16.56	71.53	16.24	4.17	2.74

**Table 7:** F statistics for the between group comparisons in the mixed models for repeated measures analysis.

	Mean Difference*	F (df = 1.119)	Cohen's d
DSF	0.91	10.09**	0.58∧∧
DSR	0.85	10.26**	0.58∧∧
DST	1.75	14.83***	0.70∧∧
TMT-A	−3.84	2.02	
TMT-B	−10.83	4.53*	−0.40∧
TMT-T	−14.67	5.05*	−0.40∧
TONI-3	3.64	1.94	
WHO-5	−2.90	0.67	

Comment to tables 4, 5, 6, and 7:

DSF = Digit Span Forward, DSR = Digit Span Reverse, DST = Digit Span Total, TMT-A = Trail Making Test Part A, TMT-B = Trail Making Test Part B, TMT-T = Trail Making Test Total, TONI-3 =  Test of non-verbal intelligence, 3rd ed., WHO-5 =  WHO Well-Being Index.

1. Significance levels.

*  =  significant at the level of 0.05.

**  =  significant at the level of 0.01.

***  =  significant at the level of 0.001.

Alpha corrected for multiple comparisons  =  0.007.

2. Cohen's d effect sizes.

∧  =  small-sized effect.

∧∧  =  medium-sized effect.

2. TMT test.

Lower scores indicate better performance.

#### ii. Missing values

We processed and analysed 1904 cognitive data points [119 participants ×8 total scores variables (obtained by summing up correct responses on individual items) per administration ×2 administrations]. Among the 1904 data points, 51 (2.6 per cent) had missing data. To prevent substantial loss of data from the data analysis owing to these missing data, and to be able to conduct a more stringent per-protocol analysis, using Cohen & Cohen procedure [35], the scores for the missing variables were plugged by deriving an individual predicted score based on an individual's actual scores on the non-missing cognitive measures. The following steps were followed to predict missing scores. All the non-missing measures were entered as independent variables in a multiple regression analysis with the dependent variable being the missing variable. Predicted scores were then computed by multiplying each variable by its Beta weight and then adding up the products plus the constant. The 51 missing values were then replaced by their corresponding predicted scores. Pre-test scores were predicted based on pre-test measures for all participants. Post-test scores were predicted separately for each group, based on the group's pre-test or post-test measurements.

## Results

### Participants

Before controlled testing began, 12 participants withdrew due to time and health constraints; within the first week, at the beginning of baseline testing, another 9 withdrew due to disinterest or health problems. Thus 119 (85%) of enrolled participants completed the study (60 in the cognitive training group and 59 in the active comparator control group). Tables 2 and 3 show that both groups were comparable on the personal and demographic attributes measured at baseline, on cognitive reserve and on engagement in challenging mental and physical activities during the length of the intervention.

### Baseline, Between-Group and Within-Group Differences

Tables 4, 5, 6 and 7 present mixed models statistics for working memory task (DSF, DSR and DST), executive control (TMT-A, TMT-B and TMT-T), non-verbal analogical reasoning (TONI 3) and life satisfaction (WHO-5) for the 119 participants (60 participants in the cognitive training group and 59 in the control group).

#### i. Groups performance at baseline

Table 4 shows that, at the onset of the study, the groups were equally competent on the selected outcomes.

#### ii. Within-group comparisons

**Figure 1:**
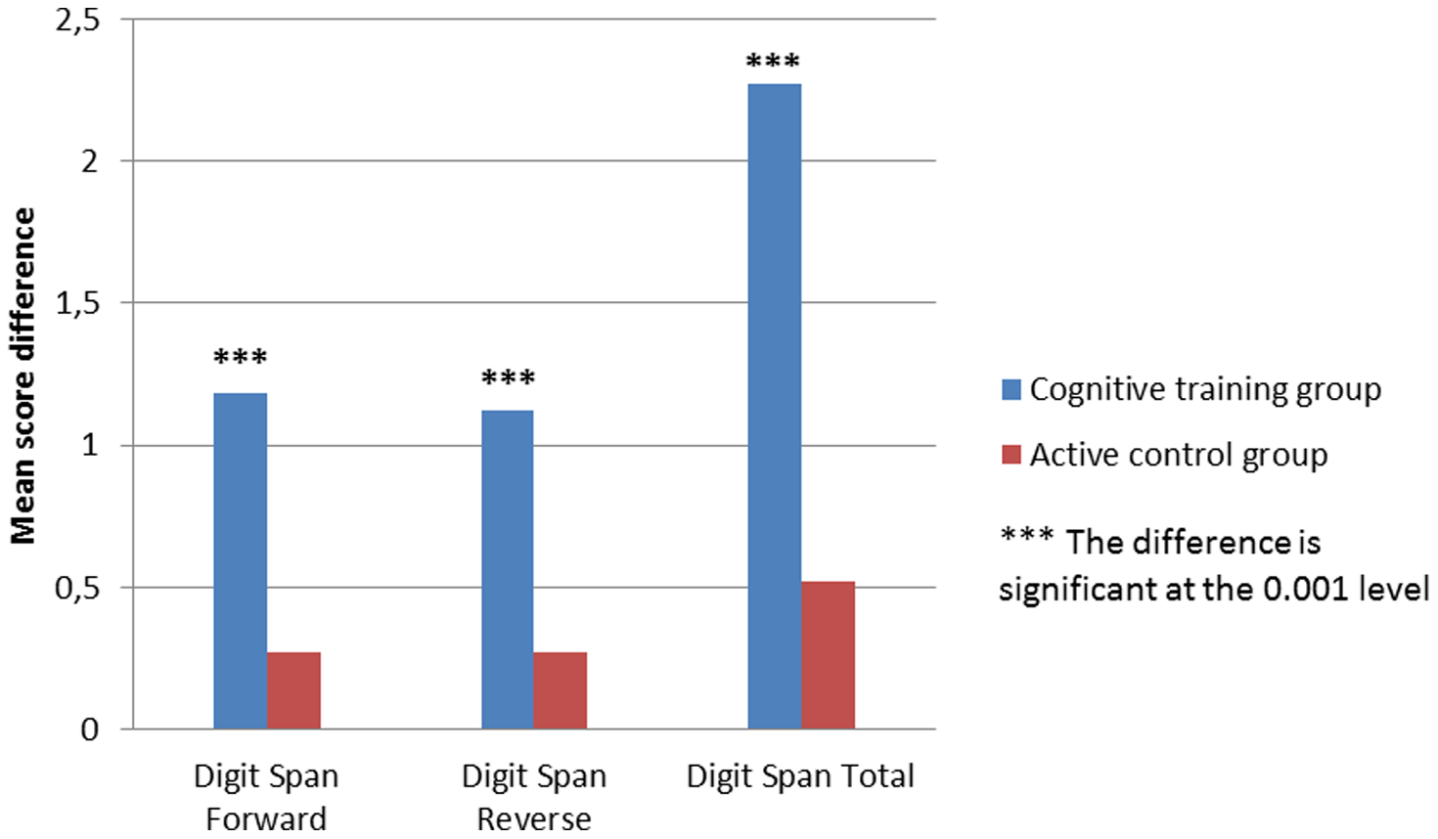
Baseline and post-training mean differences on the Digit Span.

**Figure 2:**
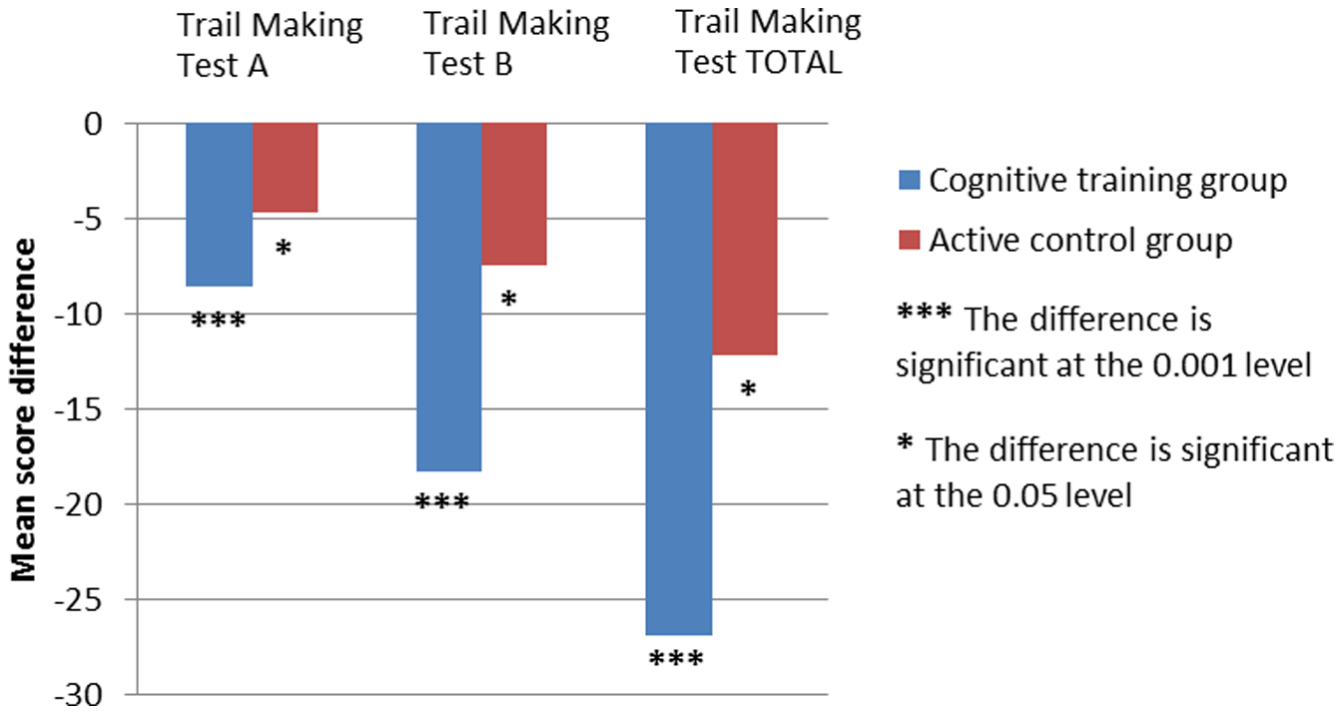
Baseline and post-training mean differences on the Trail Making Test.

**Figure 3:**
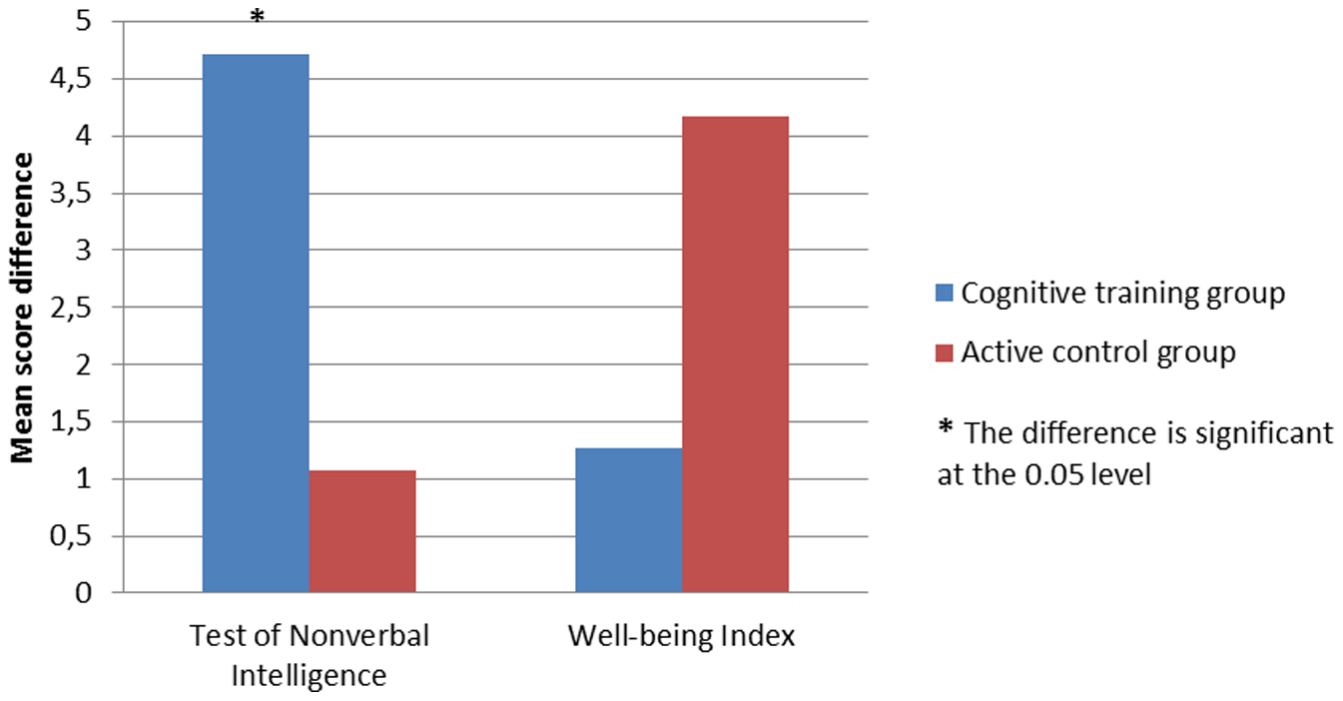
Baseline and post-training mean differences on the TONI-3 and WHO-5.

Tables 5 and 6 and Figures 1, 2 and 3 present intervention gains for each group separately. A significant effect of cognitive training was observed within the cognitive training group (Table 5) for the working memory and executive control tasks at the alpha level of 0.00625, corrected for multiple comparisons. Within the active control group (Table 6) there was no significant effect, at the corrected alpha level of 0.00625. The corrected alpha level (0.00625) was obtained by dividing the traditionally accepted alpha level (0.05) by the number of comparisons (8 comparisons).

#### iii. Between-groups comparisons

Table 7 presents the main results of the experiment: the between-groups comparisons. The table shows that, when compared to the active control group, on pre to post intervention gain, the cognitive training group showed significant improvements on DSF, DSR, DST, and on TMT-B as well as TMT-T. Using the Mean Differences and their Standard Deviations, Cohen's *d* calculated for those cognitive abilities, fell in the small to medium range (Table 7).

### System Usability

After eight weeks of cognitive training 68% of users rated the iTV system usability as “good” or “excellent”. All home users rated the iTV system as “good” or “excellent”, compared to 62% of lab users.

No significant 3-way (OUTCOME by GROUP [cognitive training vs. control group] by DEVICE [pointer vs. remote control]) interactions were observed when data were analysed to examine the contribution of pointer and remote control to DSF, DSR, DST, TMT-A, TMT-B, TMT-T or TONI-3 scores [(df = 4,12) and F  = 1.95, p = .22; F = .1.06, p = .39; F = 1.59, p = .18; F = 1.13, p = .35; F = 1.71, p = .15; F = 1.58, p = .18; F = 1.07, p = .38 respectively), suggesting that pointer and remote control were equally effective devices.

Personal diaries were collected from 104 [87.4%] participants, of which 36 (22 [44%] in the cognitive training group and 14 [26%] in the control group) indicated that using a computer represented a main challenge in their independent cognitive activities.

## Discussion

This study aimed to design, implement and test iTV-delivered, user-friendly cognitive training that might improve fluid, higher-order cognitive function in persons aged 60 years or older. We found that, when compared to an active control group, the cognitive training group improved more than the control group on well validated tests of working memory and executive control and that 67% of lab participants and 100% of home users rated the iTV system user-friendliness as “good” or “excellent”. Also, it is of special interest that none of the participants left the study after the first week of baseline testing, indicating widespread acceptance of the iTV system and activities. To our knowledge, this is the first time that an engineered iTV system has enabled older people to engage autonomously in cognitive training in laboratory and home settings and has yielded improvements on well-known and well-validated untrained cognitive measures for the cognitive training group only. The findings provide evidence for the viability and usability of TV-based cognitive training, and add to the evidence base for computer-based cognitive training induced plasticity in healthy [15], [16] and frail [18], [22] older adults, and for the significant plasticity of their prefrontal control system [4]. Older people who could benefit from cognitive enrichment but cannot use or afford a computer can easily learn how to use their television set and a pointer or remote control to engage in cognitive training, a conclusion strengthened by other published data [36] and by informal observation. Our records show that, after installation of the iTV, home users required only one brief tutorial to use the iTV cognitive training autonomously for the length of the study. Support from a partner or relative could be an important motivating factor in this context [16]. The active control group showed improvement in life satisfaction at borderline significance. This could be because they were dealing with highly meaningful materials on the personal level. The integration of mental activity with simple and ergonomic interfaces remains a considerable technical challenge [5]. Intelligent ambient assisted living environments designed to augment functionality in everyday life by the use of sensors or ubiquitous computing would not achieve expected results without acceptance on the part of elderly users. As suggested by Bureš [36], incorporating sophisticated computing principles with stress-free familiar technologies (such as TV, telephone, pointer, remote control) can help foster such acceptance.

These results may have broad societal implications. A cognitively healthy population fuels economic growth and prosperity because people remain active in society longer and put less strain on health and social care systems [37], [38]. With rising life expectancy and age as the leading risk factor for cognitive decline and dementia and in view of the computer-literacy hurdles faced by older adults, TV-based cognitive training might help to prolong personal autonomy in people who do not use computers but own or have access to TV. Unlike computer literacy, which is strongly associated with age, income and education [7], the omnipresence of the TV set means that, regardless of socio-economic status, cognitive training may eventually reach the homes of older persons to enable maintenance and growth of cognitive function.

## Acknowledgments

We thank David Cohen, Professor and Marjorie Crump Chair in Social Welfare, UCLA Luskin School of Public Affairs for his critical discussion and reading of the manuscript. Authors would like to thank all Vital Mind project members from the Philips Consumer Products Innovative Lab, Czech Technical University in Prague, University of Genoa, Czech TV, University of Dundee, University of Hradec Králové, Goldsmith College in London, and CogniFit, the coordinator, for their valuable contribution to the project.

## Supporting Information

Data S1 *VitalMind_DATA_S1.sav* comprises anonymised data gathered during the experiment.
(SAV)

Plugged Data S1 *S_VitalMind_PLUGGED_DATA_S1.sav* contains adjusted data used for analyses (as described in the section Missing values).
(SAV)

Cognitive Training Programme S1 *Cognitive_training_programme_S1.docx* lists names and descriptions of tasks in the cognitive training programme.
(DOCX)
