# Supplementary material for: Novel Television-Based Cognitive Training Improves Working Memory and Executive Function
Source: PLoS One. 2014 Jul 3;9(7):e101472. doi: 10.1371/journal.pone.0101472 (PMC4081563; doi:10.1371/journal.pone.0101472)
Supplement: Cognitive Training Programme S1 — Cognitive_training_programme_S1.docx lists names and descriptions of tasks in the cognitive training programme. (DOCX) [file pone.0101472.s003.docx]

Names and descriptions of tasks in the cognitive training programme.

1. *The Common Factor*: A name of semantic categories (for example, "transport") are displayed, and various objects are presented either as pictures, spoken words or written words. The user has to decide if each of the objects 'Belongs' or 'Does Not Belong' to the semantic category.

*2. My Name Is Alice:* An image of an object is displayed on the screen for a short period of time. After the image disappears, four letters are displayed, and the task is to choose the letter, among the four, which is the first letter of the object's name.

3. *Up, Down And Around*: Four arrows are displayed in four positions: Up, Down, Left and Right. The users are asked to click on the arrows according to instructions in either the auditory or the visual instructions modes. Distractions, which the users need to ignore, are also embedded in this task.

4. *CrissCross*: Two different tasks are given. After training each of them separately, the screen is divided into two, and one part is activated at a time, displaying one of the tasks.

After a few seconds the other side is activated, and the users need to switch to the other task as quickly as possible and to continue switching each time the other task is activated. One task is a divided attention task: A ball is moving toward a wall. If the colour of the ball matches the colour of the wall it is going to hit, the users have to refrain from responding. But, if the ball's colour doesn't match the colour of the wall towards which it is moving, the users have to click on that wall, so the colour will change to that of the ball. The second task is a tracking task. The user places the computer mouse cursor on a moving ball and needs to keep it there, being as accurate as possible.

5. *Chain Code*: A series of digits are presented either in the visual or in the auditory mode. The task is to remember the exact sequence of digits. Both immediate and delayed retrieval are trained.

6. *What Happened To My Mouse*: This task trains and challenges the computer 'mouse'skills. The mouse begins to behave strangely and move in unexpected directions. The users have to control the mouse and click on puzzle pieces in numerical order as quickly as possible.

7. *Pick A Pair*: This is a learning task. The users can choose from three categories: Country flags, famous people and monuments around the world. In each of the categories pairs are presented for leaning.

8. *Music and Rhythm*: In this task, the users first familiarise themselves with the sounds of 10 musical instruments. Later, they are asked to identify the instruments by their sounds, and also to identify some tempo patterns.

9. *Bell Boy*: Two elevators are displayed. The users get a stimulus from each elevator, either visual or auditory. The task is to estimate which stimulus is longer.

10. *Inside And Outside*: Pictures are displayed, one at a time, in the centre of the screen.

Smaller pictures are also displayed in different locations in the outer circle. The task is to spot the pictures in the outer circle that are similar to the pictures in the centre.

11. *Keep the Track*: A route is displayed, with a ball at the starting point. The ball starts moving and the task is to track it with the mouse cursor, always moving at the same speed as the ball. Occasionally the ball and also the route disappear. The task is to continue moving the cursor at the same speed/direction as the ball.

12 . *Supermind*: The computer chooses a secret code, consisting of sets of two, three or four digits or coloured golf-balls. The task is to break the code by finding out the exact locations of each item in the set.

13. *Morning Time*: Windows, arranged at random on the screen, are opened momentarily one after the other. The task is to follow the exact sequence in which the windows were 28 opened. The task evolves as the user remembers longer sets.

14. *Hot Air Balloon*: A hot air balloon flies in the sky. Along its way, it lands on different clouds. The task is to remember its exact route.

15. *Two in One*: Two rooms with different coloured walls are displayed. A coloured ball is moving on a collision course with a wall in each room. The task is to match the colour of the wall to the colour of the ball, working simultaneously on the two rooms.

16. *Who and Where*: Pictures are displayed for a short time. The task is to remember the exact pictures in their exact locations.

17. *Hidden Words*: An object appears in the lower left corner of the screen. The task is to find the name of this object spelled out in the letter box.

18. Picture Arrangement: A distorted picture is displayed. The task is to rearrange the picture by moving the pieces to their correct position.

19. *Picasso*: On the right side of the screen an abstract design is displayed for a short time only. The left side of the screen displays squares and triangles. The task is to reconstruct the abstract design, using the shapes from the left side of the screen.

20. *Of Balls and Pictures*: There are two tasks. First, each task is presented separately and then they are combined in order to perform the two tasks simultaneously. In the 'Balls' task balls are moving on a grid, and the task is to prevent them from colliding. When two balls move towards the same intersection, the task is to click on that intersection, as quickly as possible, before the balls collide. In the 'Pictures' task, pictures are displayed at the four corners of the screen. The task is to identify similar pictures, and press the 'Space bar' with the hand that is not controlling the computer mouse.

21. *Fast-Moving Objects*: Objects appear and disappear quickly from either the left or the right side of the screen. The task is to identify them.
